# Strabismus

**Published:** 1885-08-20

**Authors:** W. L. Bullard

**Affiliations:** Columbus, Ga.


### STRABISMUS.

BY W. L. BULLARD, OF COLUMBUS, GA.

According to Tonders, squint is defined as a deviation in the direction of eyes, in consequence of which the two yellow spots receive images from different objects.

The cases mostly met with are either convergent or divergent. We sometimes, however, meet with cases in which the deviation is solely upwards or downwards. In monocular strabismus, the squinting eye is generally associated with impairment of sight. In alternating or binocular strabismus the patient can fix with either eye, but is not able to focus the two together at the same object. In the alternating form, the sight of both eyes are about the same.

I think the largest [illegible]ber of squint cases are caused by some anomaly in the re[illegible]f the eyes, as hypermetropia and myopia. It is also in[illegible]m defective vision in one eye, no matter whether it be from opacities of the cornea or from great differences in the refraction of both eyes. In partial or complete paralysis of the nerve-supply to the ocular muscles, squint is also sometimes a sequel. Most all cases of convergent or internal strabismus are dependent on hypermetropia. It generally commences in early life. A patient who is hypermetropic has always to use an excessive amount of accommodation in order to see objects clearly, and the act of accommodation is naturally associated with that of convergence. This being the case, we meet with patients who attribute their trouble to different causes. Some will say that it comes on during convalescence from an attack of fever; others will date it from a severe case of "sore eyes," while a few will say that their trouble came from excessive study after commencing school. Now, from whatever cause the ocular muscles are weakened, and, in consequence of which, are not able to overcome the accommodation necessary to render the eyes emmetropic, consequently the visual lines cross between the patient and the object looked at; hence, the patient, or child, is cross-eyed. Divergent strabismus most commonly occurs in myopic eyes; yet it is sometimes found in both emmetropic and hypermetropic eyes.

Since writing the above, I have been consulted by Miss B——, eighteen years old, for "weak eyes," with divergent squint. With the right eye, vision is $\frac{2}{200}$, left $\frac{xx}{200}$; near vision, J. No. 18. On examination with ophthalmoscope, I find the trouble to be choroiditis diseminata. In the right eye, the disease is located at the macula lutea, and the retina has already suffered, or undergone some atrophic change. Now, this young lady is 2 D. hypermetropic, yet she has divergent squint of right eye caused from a *chronio-retinitis centralis*.

In the treatment of concomitant squint, whether it be convergent or divergent, the refraction of each eye should be carefully examined in all cases, and correcting glasses prescribed. The refraction being properly corrected, oftentimes intermittent strabismus is removed without operation. When the squint is permanent, in addition to the optical correction, an operation must be performed; and if the errors of refraction is excessive, proper glasses should be adjusted and constantly worn for weeks—I might say for months—subsequent to the tenotomy.

